# An interactive network of alternative splicing events with prognostic value in geriatric lung adenocarcinoma via the regulation of splicing factors

**DOI:** 10.1042/BSR20202338

**Published:** 2020-10-16

**Authors:** Yidi Wang, Yaxuan Wang, Kenan Li, Yabing Du, Kang Cui, Pu Yu, Tengfei Zhang, Hong Liu, Wang Ma

**Affiliations:** 1 Department of Oncology, The First Affiliated Hospital of Zhengzhou University, Zhengzhou 455002, Henan, China; 2 Shangqiu Medical College, Shangqiu 476000, Henan, China

**Keywords:** alternative splicing, geriatric, lung adenocarcinoma, prognostic index models, splicing factors

## Abstract

Alternative splicing (AS), an essential process for the maturation of mRNAs, is involved in tumorigenesis and tumor progression, including angiogenesis, apoptosis, and metastasis. AS changes can be frequently observed in different tumors, especially in geriatric lung adenocarcinoma (GLAD). Previous studies have reported an association between AS events and tumorigenesis but have lacked a systematic analysis of its underlying mechanisms. In the present study, we obtained splicing event information from SpliceSeq and clinical information regarding GLAD from The Cancer Genome Atlas. Survival-associated AS events were selected to construct eight prognostic index (PI) models. We also constructed a correlation network between splicing factors (SFs) and survival-related AS events to identify a potential molecular mechanism involved in regulating AS-related events in GLAD. Our study findings confirm that AS has a strong prognostic value for GLAD and sheds light on the clinical significance of targeting SFs in the treatment of GLAD.

## Introduction

Cancer morbidity and mortality rates are rapidly increasing worldwide. Lung cancer is the most common cause of cancer-related mortality [1]. In 2018, the numbers of new cases and deaths globally for lung carcinomas were 2.1 million (11.6% of the total cancer cases) and 1.8 million (18.4% of total cancer deaths), respectively [2]. In the new World Health Organization (WHO) classification, non–small cell lung carcinomas (NSCLCs) are classified into squamous cell carcinomas, adenocarcinomas, large cell carcinomas, and mixed cell carcinomas. Among them, adenocarcinoma is the most common type, accounting for approximately 60% of NSCLCs [3]. According to the latest statistics from the American Cancer Society, lung cancer mainly occurs in the elderly. Most people diagnosed with lung cancer are 65 or older; very few people are diagnosed under 45 years of age. The average age at diagnosis is approximately 70 [1]. Considering that elderly patients always have more complications and poor prognosis, many clinical trial inclusion criteria exclude elderly patients. Thus, more attention should be paid to geriatric patients. A study analyzing the genetic characteristics of 184 patients with lung adenocarcinoma showed that distinctive genetic profile including the Kristen rat sarcoma viral oncogene, serine/threonine kinase 11 (*STK11*), and epidermal growth factor receptor (*EGFR*) exon 20 mutation were common in the older patient group. However, *EGFR*/tumor protein 53 (*TP53*) mutations, anaplastic lymphoma kinase (*ALK*), and human epidermal growth factor receptor 2 (*HER2*) genetic alterations were more prevalent in younger patients [4]. Currently, tumor biomarkers, tumor stages, and molecular markers are common indicators that predict the prognosis of patients with lung adenocarcinoma (LUAD) [5–7]. However, the number of biomarkers that can be used clinically is limited and no prognostic model was built exclusively for elderly patients. Therefore, novel and effective prediction methods are needed to predict the prognosis of GLAD.

RNA splicing is a complex and sophisticated process used to generate mature mRNAs by removing introns and joining exons. This process is highly controlled by the spliceosome [8]. The spliceosome consists of more than 300 proteins and five small nuclear RNAs (snRNAs), U1, U2, U4, U5, and U6. Spliceosome and associated RNA splicing factors (SFs) are involved in the regulation of alternative splicing (AS) events [9]. AS is an essential process for regulating gene expression and ensuring proteome diversity [10]. AS can increase the diversity of mRNAs through seven types of splicing events: alternate acceptor site (AA), alternate donor site (AD), alternate promoter (AP), alternate terminator (AT), retained intron (RI), mutually exclusive exons (ME), and exon skip (ES) [10]. Abnormal AS events can produce aberrant protein isoforms, which may contribute to the development of tumors [11–13]. Genome-wide studies have publicized more than 15,000 tumor-related splice variants in 27 types of cancers [14]. David et al. showed that splicing events were related to tumorigenesis and to progression, including angiogenesis, metastasis, and apoptosis [15]. Other studies have indicated that AS changes are involved in the prognosis of different tumors, such as prostate cancer, papillary thyroid cancer, and uterine corpus endometrial cancer [16–18]. These studies reported an association between AS events and tumorigenesis. However, systematic survival analyses of AS in GLAD are lacking. Studies have revealed specific roles for SFs in lung cancer development [19], and some of these studies have suggested that SFs can regulate the aberrant process of AS with effects on the occurrence and development of lung cancer [20]. Multiple studies have indicated that AS events have diagnostic value and could be considered potential drug targets [21–24]. Given the high incidence of splicing defects in lung adenocarcinoma, the potential connection between SFs and AS events in GLAD deserves further exploration and supporting evidence.

The Cancer Genome Atlas (TCGA) is a project that classifies the major cancer-related genomic alterations [25]. Currently, a single gene or molecular marker cannot accurately predict the progression of the disease. With the development of high-throughput sequencing technology, many studies have established predictive models that pay closer attention to the interaction of multiple genes [26–28]. However, no study currently has provided evidence supporting the prognostic value of AS events in GLAD. Thus, for the present study we downloaded splicing data from TCGA of geriatric patients with LUAD to build the GLAD prognosis model. A splicing network between SFs and survival-related AS events was established to demonstrate potential molecular mechanisms involved in regulating AS-related events in GLAD.

## Materials and methods

### Data acquisition and organization

The clinical information of 251 elderly patients with LUAD (age 65–89) and mRNA expression data were downloaded from the TCGA Genomic Data Commons (GDC) (https://portal.gdc.cancer.gov/) database. Cancer-related AS events were selected in 59 normal controls and 513 tumor tissues from the TCGA SpliceSeq database (https://bioinformatics.mdanderson.org/TCGASpliceSeq/). In addition, we obtained the percent-sliced-in (PSI) values for seven types of AS events from RNA-seq to quantify them in GLAD patients.

### Statistical analysis

**Figure 1:**
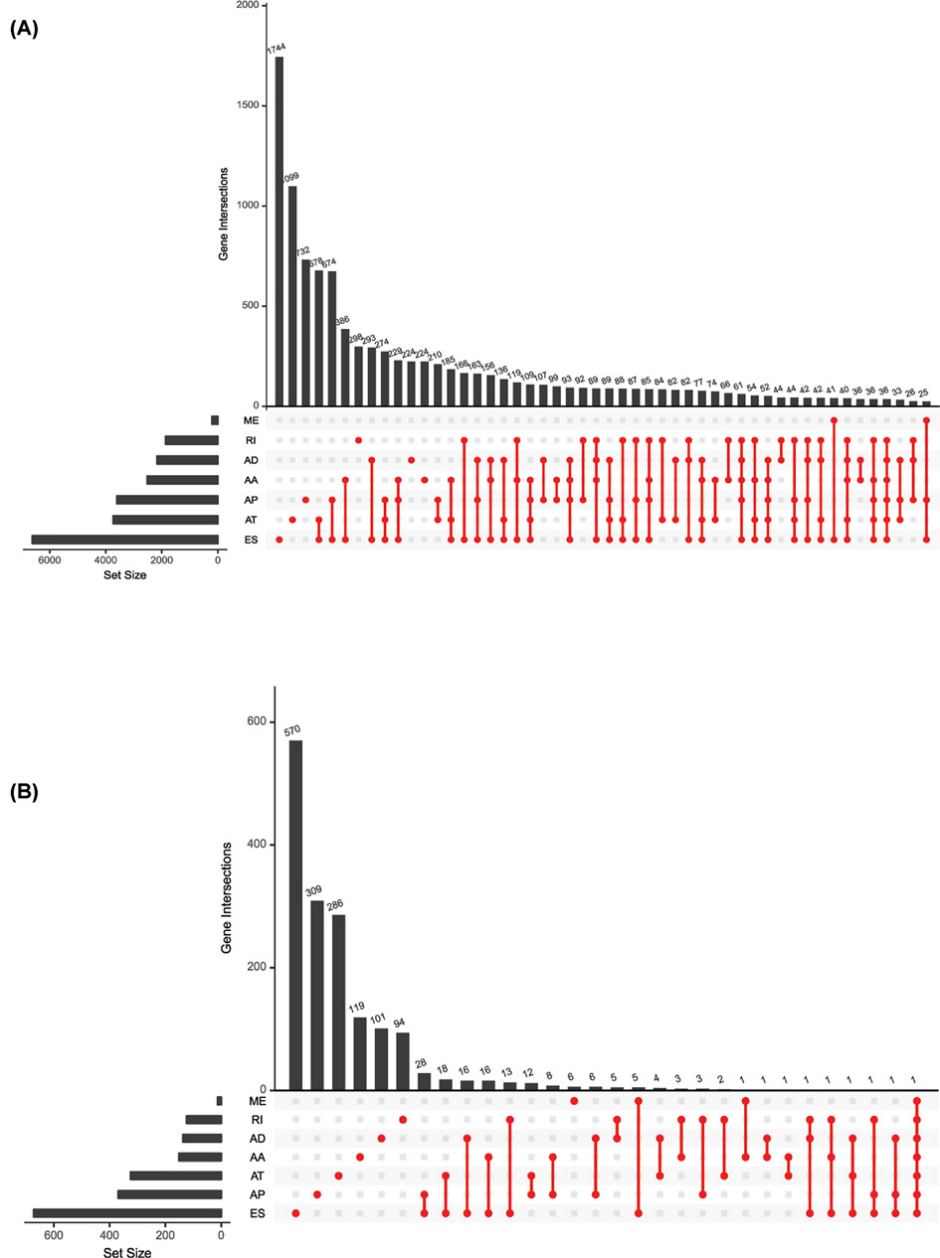
UpSet plots of alternative splicing (AS) events in geriatric lung adenocarcinoma (GLAD) (**A**) UpSet plots of AS events in GLAD. The horizontal axis represents the number of genes in each AS type. The vertical axis represents the number of genes for one or several splicing types. (**B**) UpSet plots of survival-related AS events in GLAD.

Univariate Cox regression analyses were conducted to select survival associated AS events. UpSet, a novel technique was used to visualize intersecting sets [29]. Specific splicing events were illustrated in the UpSet plots generated by the R package (Figure 1A,B). The R package was also used to create Volcano plots and Bubble charts to reveal the prognostic related AS events. Further, univariate analyses and multivariate analyses were performed to explore independent survival-related prognostic factors.

### Construction of the prognostic index models

Univariate Cox regression analyses were conducted to select survival associated AS events (*P*<0.05). Least absolute shrinkage and selection operator (LASSO) regression analysis was used to screen and eliminate genes with high correlation to construct a credible prognostic index model. Genes with high survival correlations selected by LASSO regression were used to construct PI models. In addition, risk curves were generated using the R package based on the risk score. We calculated the risk value for each case based on the following formula:

$$\text{Risk}\;\text{score}=\sum_{i}^{n}\text{PSI(i)}\times \text{C(i)}$$

where PSI is the percent that was spliced in to indicate AS event changes [30], and *C* is the regression coefficient.

### Evaluation of the prognostic value of the prognostic index models

Based on the median value of the risk score calculated by the PI model, cases were divided into high-risk and low-risk groups. Kaplan–Meier curve (K-M) analysis was used to describe survival probabilities. To certify the reliability of the model in predicting prognosis, the survival receiver-operator characteristic (ROC) package in R was used to calculate the area under the curve (AUC) of the ROC curve for each model. Models with AUC > 0.7 were more effective models. We then substituted data from non-elderly lung adenocarcinoma patients (age 33–64) into the PI models to demonstrate that AS events differed between elderly and non-elderly lung adenocarcinoma patients. Differences that may arise from the differential expression of genes in elderly and non-elderly patients.

### Correlation network between alternative splicing and splicing factors

SFs played a significant role in regulating splicing events. We obtained the information regarding SFs from the database SpliceAid2 (http://www.introni.it/splicing.html). The mRNA expression of SFs in geriatric lung adenocarcinoma was downloaded from the TCGA database. Survival-related SFs were screened by univariate Cox regression analysis. Pearson correlation analysis was used to analyze the correlation between survival-related SFs and AS events with independent predictive significance (|correlation coefficient| > 0.6, *P*<0.001). We explored the correlation network of survival-associated SFs and prognosis-associated AS events to further understand the underlying molecular mechanism of AS events in GLAD. Cytoscape v3.7.1 software was used to visualize network data such as genetic, protein–DNA, and protein–protein interactions [31] and generated a potential regulatory network between AS and SFs. Then, based on the R package, the Gene Ontology (GO) terms and Kyoto Encyclopedia of Genes and Genomes (KEGG) pathways were used to assess the functions associated with the most significant prognosis-related AS events.

## Results

### Clinical characteristics

In the present study, cancer-related AS events were selected in 59 normal controls and 513 tumor tissues from the TCGA SpliceSeq database. A total of 251 patients were included in our analysis. We processed the clinical information and TCGA SpliceSeq files of geriatric patients with LUAD. Univariate analyses and multivariate analyses were performed to explore independent survival-related prognostic factors (Table 1). In the univariate Cox hazard analyses, the TNM stage (hazard ratio (HR) = 1.653, 95% CI: 1.300–2.103; *P*<0.001), tumor stage (HR = 1.64, 95% CI: 1.206–2.233; *P*<0.05), lymph node metastasis (HR = 2.199, 95% CI: 1.625–2.977; *P*<0.001), and risk score of eight PI models were significantly correlated with the survival time of elderly patients with LUAD. However, no significant correlations were observed between survival time and age, sex, or distant metastasis. According to the multivariate cox hazard analysis, only the risk score of eight PI models significantly correlated with the survival time of geriatric patients with LUAD. This demonstrated that the PI model could be used as an independent prognostic factor to predict the prognosis of LUADs in geriatric patients.

**Table 1:** Univariate and multivariate Cox regression analysis of clinical variables and eight prognostic index models of lung adenocarcinoma in geriatric patients

Clinical variable	Group	Univariate		Multivariate	
		HR (95%CI)	*P*-value	HR (95%CI)	*P*-value
Age	<75	1.022 (0.970–1.076)	0.413	1.001 (0.955–1.068)	0.726
	≧75				
Sex	Male	0.980 (0.586–1.639)	0.938	0.832 (0.485–1.426)	0.503
	Female				
Stage-TMN	I-II	1.653 (1.300–2.103)	4.25E-05	1.112 (0.437–2.833)	0.824
	III-IV				
T	T1-T2	1.641 (1.206–2.233)	0.002	1.167 (0.747–1.825)	0.498
	T3-T4				
M	M0	1.023 (0.308–3.403)	0.97	0.998 (0.069–14.501)	0.999
	M1				
N	N0	2.199 (1.625–2.977)	3.33E-07	1.738 (0.776–3.892)	0.178
	N1				
Risk score of AA	High-risk	1.096 (1.066–1.126)	4.62E-11	1.082 (1.051–1.115)	1.81E07
	Low-risk				
Risk score of AD	High-risk	1.122 (1.085–1.161)	2.50E-11	1.100 (1.060–1.141)	<0.001
	Low-risk				
Risk score of AP	High-risk	1.195 (1.139–1.253)	2.21E-13	1.169 (1.111–1.230)	1.54E09
	Low-risk				
Risk score of AT	High-risk	1.155 (1.108–1.205)	2.13E-11	1.125 (1.072–1.180)	1.75E-06
	Low-risk				
Risk score of ES	High-risk	1.102 (1.071–1.134)	2.37E-11	1.096 (1.058–1.135)	2.83E-07
	Low-risk				
Risk score of ME	High-risk	1.233 (1.146–1.328)	2.50E-08	1.254 (1.153–1.363)	1.07E-07
	Low-risk				
Risk score of RI	High-risk	1.065 (1.041–1.089)	2.84E-08	1.056 (1.031–1.081)	5.05E-06
	Low-risk				
Risk score of ALL	High-risk	1.071 (1.050–1.095)	1.10E-10	1.062 (1.038–1.087)	1.79E-07
	Low-risk				

Abbreviations: AA, alternate acceptor site; AD, alternate donor site; AP, alternate promoter; AT, alternate terminator; ES, exon skip; HR, hazard ratio; ME, mutually exclusive exons; RI, retained intron.

### Survival-associated alternative splicing events

**Figure 2:**
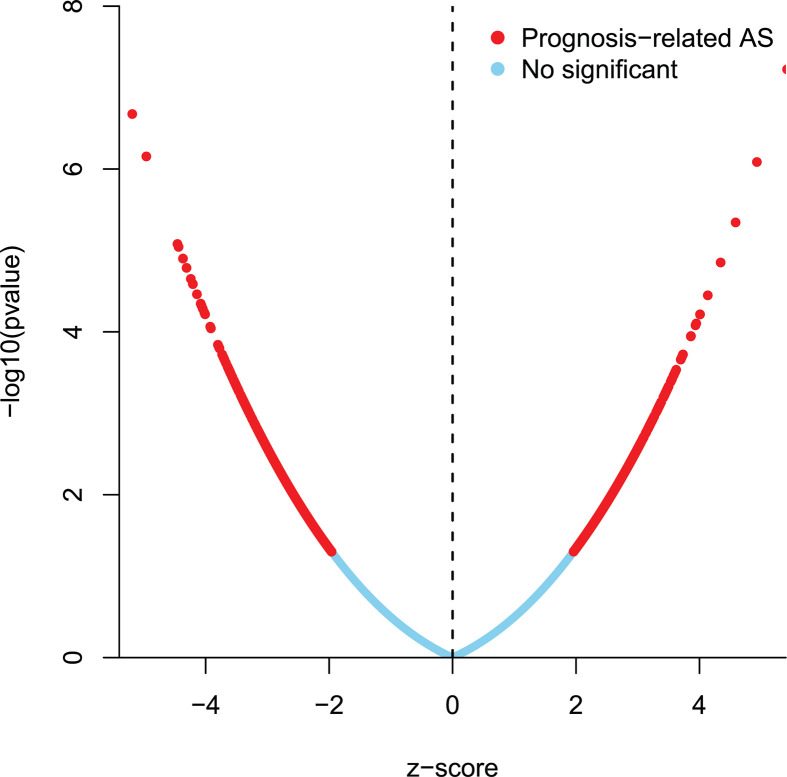
Volcano plot of AS events (*P*<0.05) The red dots indicate prognosis-related AS events; the blue dots indicate the events with no significance.

**Figure 3:**
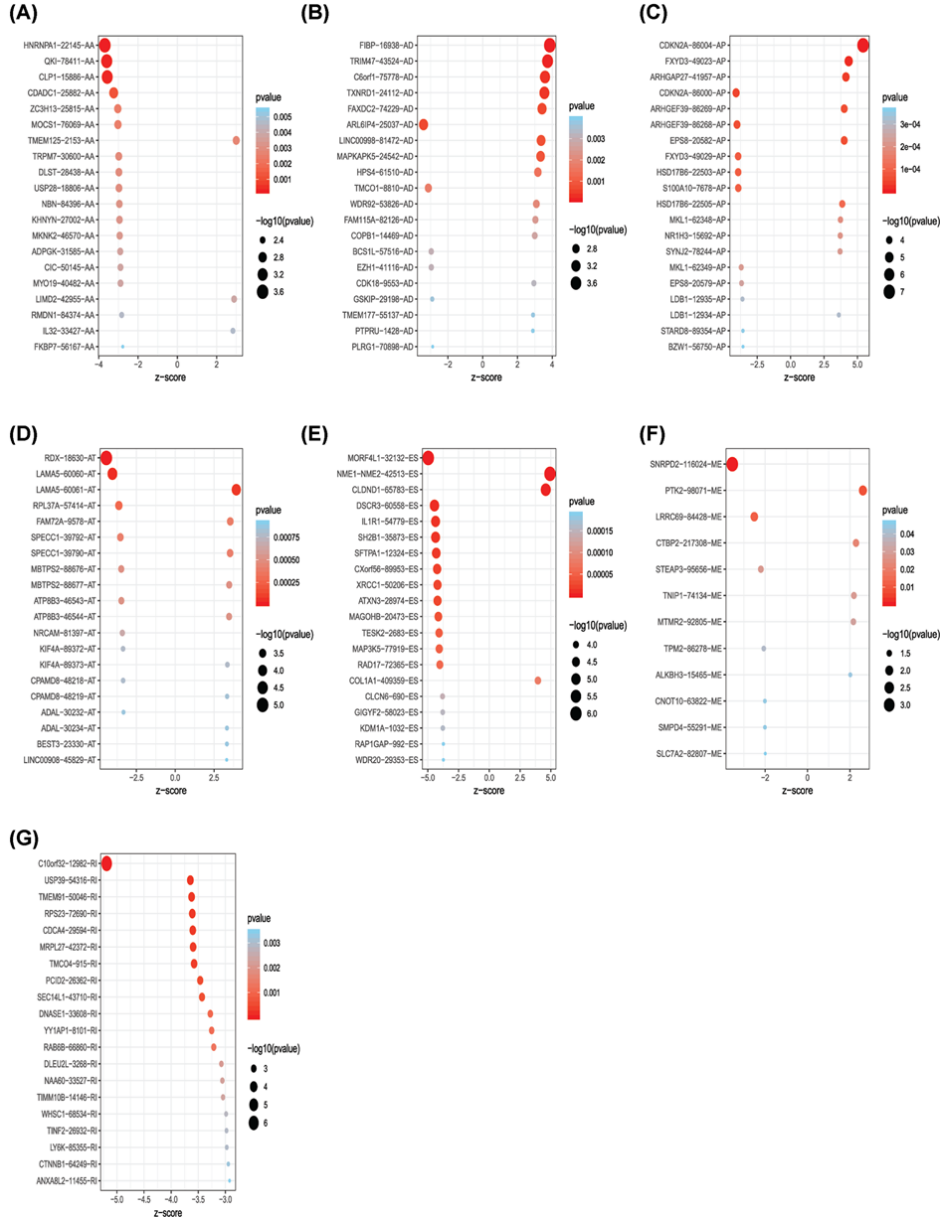
Bubble plots of the most significant genes with seven types of survival-associated alternative splicing events The ID of the specific AS event is displayed on the vertical axis. Events with greater significance are represented by larger circles and are colored in red. (**A**) 20 prognosis-related AA events; (**B**) 20 prognosis-related AD events; (**C**) 20 prognosis-related AP events; (**D**) 20 prognosis-related AT events; (**E**) 20 prognosis-related ES events; (**F**) 12 prognosis-related ME events; (**G**) 20 prognosis-related RI events.

The intersections of genes and AS events in GLAD were described using the following UpSet plot (Figure 1A,B), which indicated that a single gene may be involved in different AS events. Of seven types of AS events, ES was the most common type. A total of 16,793 ES events were observed in 6475 genes, of which only 1744 genes were involved in the ES event. We also identified 3559 AA events in 2306 genes, 3057 AD events in 1710 genes, 8992 AP events in 3398 genes, 8546 AT events in 3522 genes, 220 ME events in 65 genes, and 2781 RI events in 1681 genes (Figure 1A). After combining AS data and survival data, a total of 2381 survival-related AS events in 1633 genes were reported through the univariate Cox regression analysis (*P*<0.05), including 158 AA events in 151 genes, 145 AD events in 137 genes, 580 AP events in 369 genes, 527 AT events in 325 genes, 825 ES events in 672 genes, 12 ME events in 13 genes, and 124 RI events in 124 genes (Figure 1B). The distribution of AS events correlated with patient survival are displayed in the Volcano plot (*P*<0.05) shown in Figure 2. Furthermore, the bubble charts in Figure 3 revealed the 20 most significant prognostic-related AS events; however, only 12 prognostic related ME events were included. As shown, each AS event is defined by a unique code. For example, for the code *ATAD3A*-176-AA, *ATAD3A* is the name of gene, 176 is the sequence number of the splicing event in the TCGA database, and AA is the splicing type.

### Prognostic value of prognostic indexes in survival analysis

**Figure 4:**
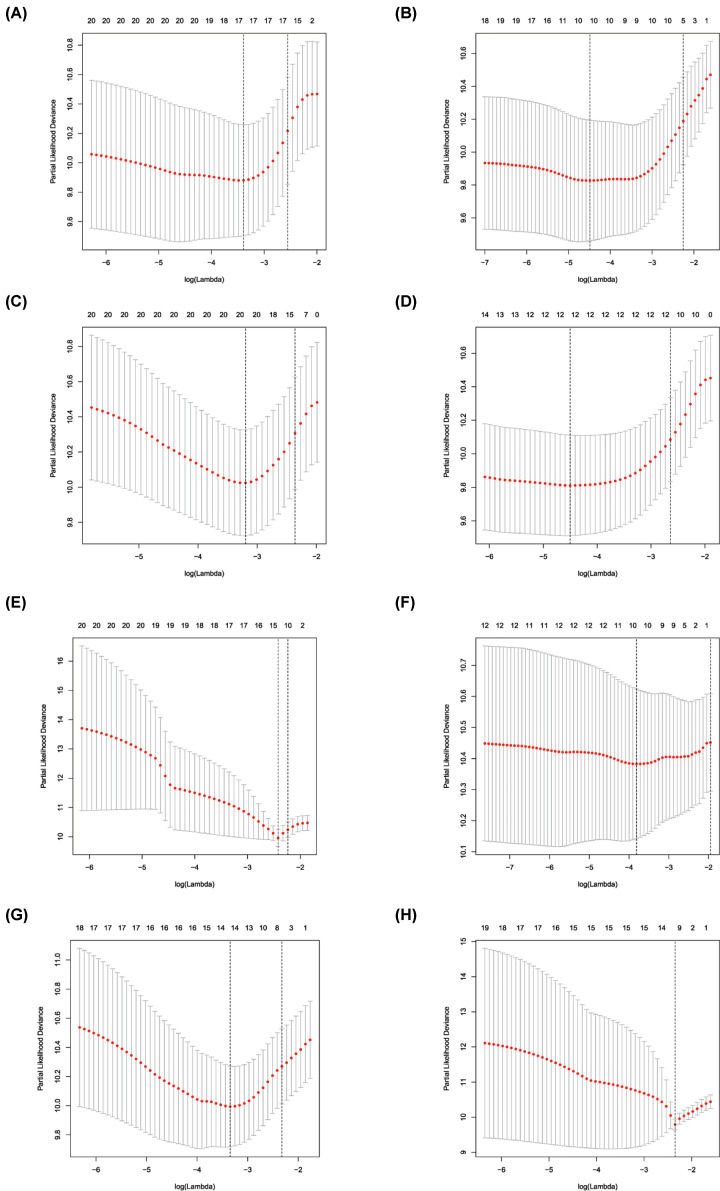
Construction of prognostic signatures based on least absolute shrinkage and selection operator (LASSO) Cox analysis (**A–H**) The lowest point of the ordinate is the minimum point of the cross-validation error.

**Figure 5:**
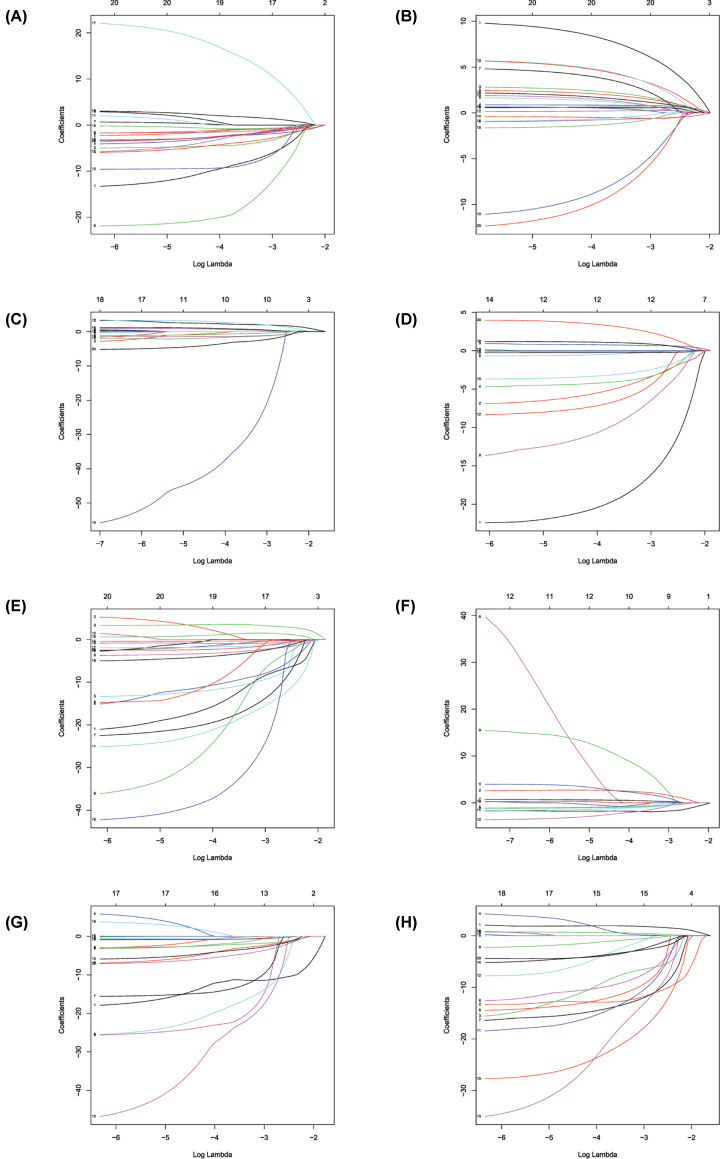
LASSO COX analysis of seven types of events (**A–H**) The horizontal axis represents the Log Lambda. The vertical axis represents the coefficients. As the value of the Log Lambda increased, the coefficient approached 0.

**Figure 6:**
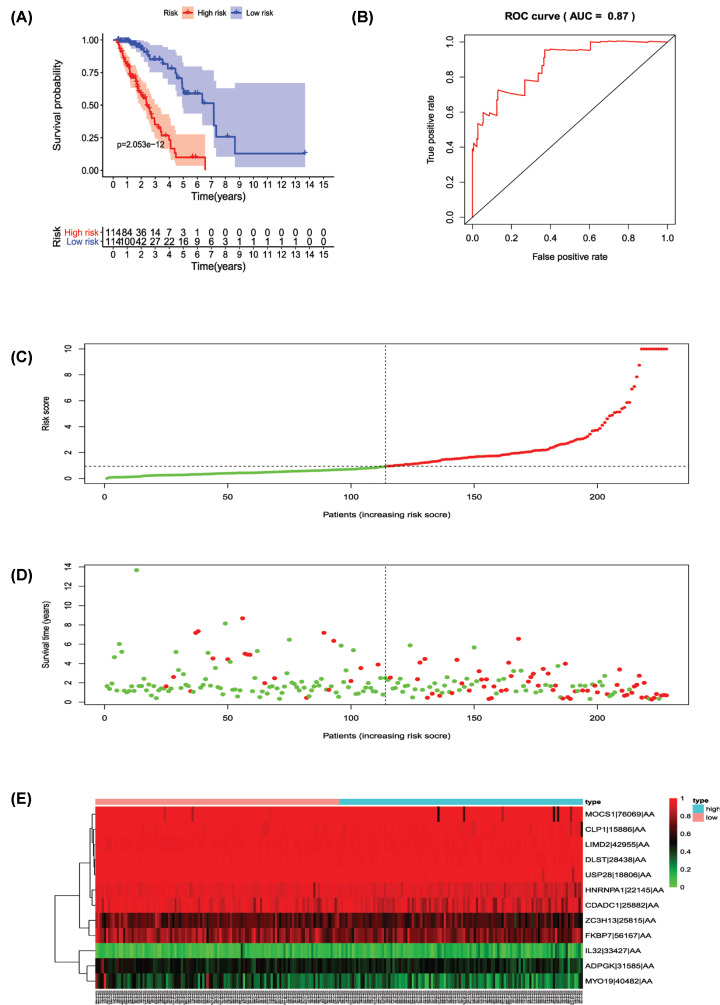
Analysis of the prognostic index (PI)-AA The 12 prognosis-associated events of AA were selected by multivariate Cox regression analysis to construct the PI-AA model. (**A**) The risk curve of the PI evaluating the survival time of geriatric patients with LUAD stratified by the median PI value into a low-risk and high-risk groups. (**B**) Time-dependent receiver-operator characteristic curve of the PI for predicting the tumor status. (**C**) PI value curve for geriatric patients with LUAD. (**D**) Survival conditions and survival time of GLAD patients distributed according to risk score (green dots represent survivors; red dots represent deaths). (**E**) Heat map indicating the relationship between the PSI value of the AS events and the risk score.

**Figure 7:**
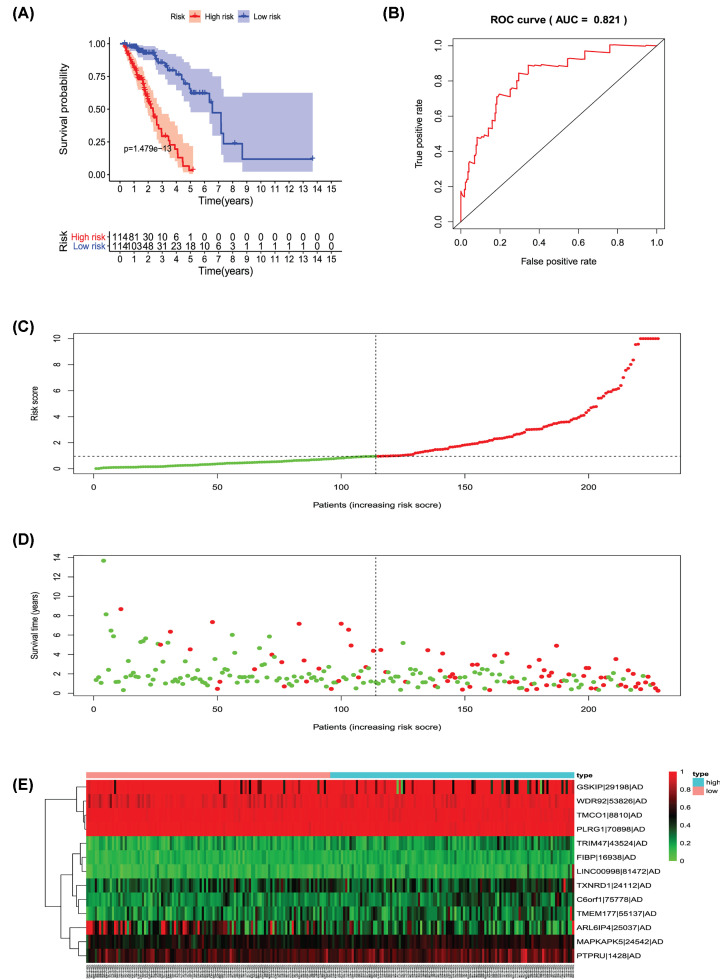
Analysis of the PI-AD Thirteen prognosis-associated events of AD were selected by multivariate Cox regression analysis to construct the PI-AD model. (**A**) The risk curve of the PI for evaluating the survival time of geriatric patients with LUAD stratified by the median PI value into a low-risk and high-risk group. (**B**) Time-dependent receiver-operator characteristic curve of PI for predicting the tumor status. (**C**) PI value curve for geriatric patients with LUAD. (**D**) Survival conditions and survival time of GLAD patients distributed according to risk score (green dots represent survivors; red dots represent deaths). (**E**) Heat map indicating the relationship between the PSI value of the AS events and the risk score.

**Figure 8:**
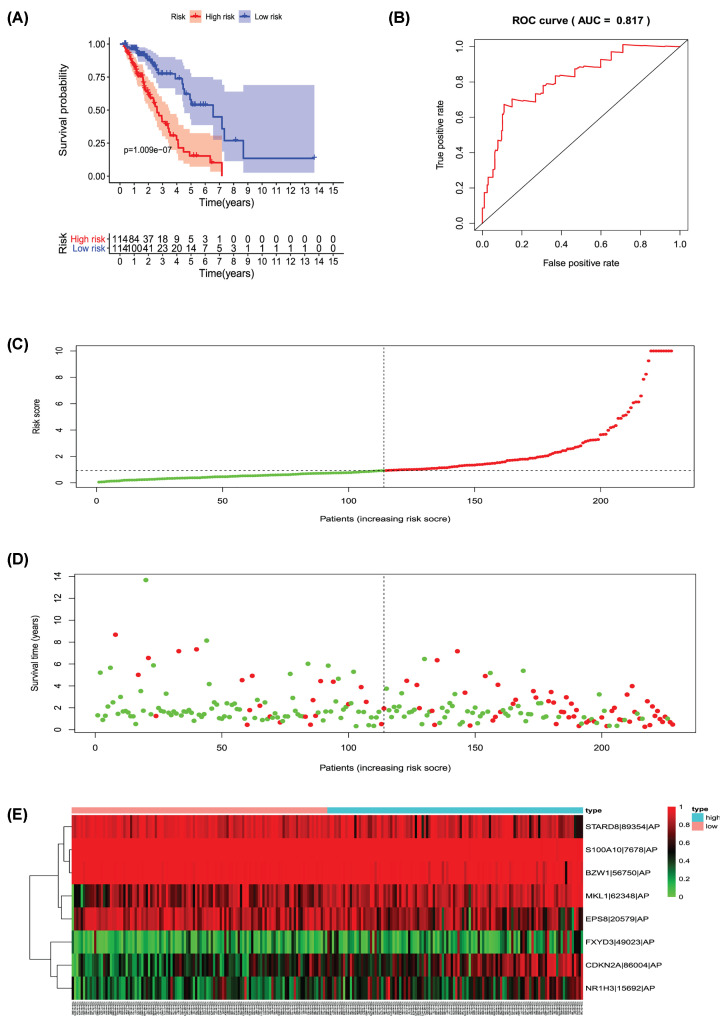
Analysis of the PI-AP The eight prognosis-associated events of AP were selected by multivariate Cox regression analysis to construct the PI-AP model. (**A**) The risk curve of the PI for evaluating the survival time of geriatric patients with LUAD stratified by the median PI value into low-risk and high-risk groups. (**B**) Time-dependent receiver-operator characteristic curve of PI for predicting the tumor status. (**C**) PI value curve for geriatric patients with LUAD. (**D**) Survival conditions and survival time of GLAD patients distributed according to risk score (green dots represent survivors; red dots represent deaths). (**E**) Heat map indicating the relationship between the PSI value of the AS events and the risk score.

**Figure 9:**
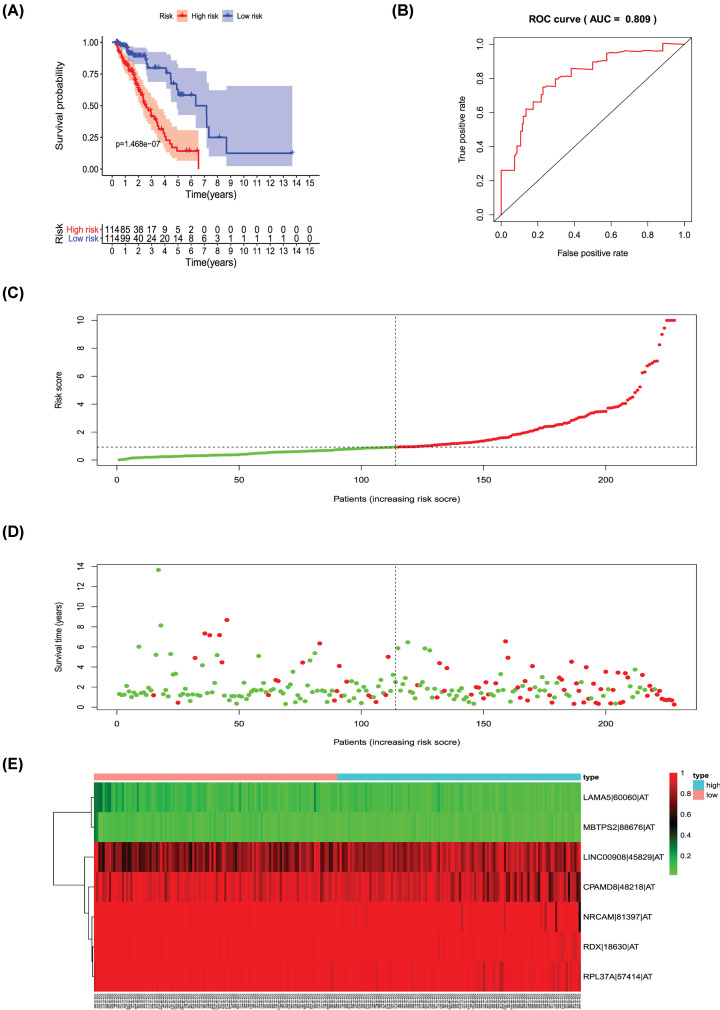
Analysis of the PI-AT The seven prognosis-associated events of AT were selected by multivariate Cox regression analysis to construct the PI-AT model. (**A**) The risk curve of PI for evaluating the survival time of geriatric patients with LUAD stratified by the median PI value into a low-risk and high-risk groups. (**B**) Time-dependent receiver-operator characteristic curve of PI for predicting the tumor status. (**C**) PI value curve for geriatric patients with LUAD. (**D**) Survival conditions and survival time of GLAD patients distributed according to risk score (green dots represent survivors; red dots represent deaths). (**E**) Heat map indicating the relationship between the PSI value of the AS events and the risk score.

**Figure 10:**
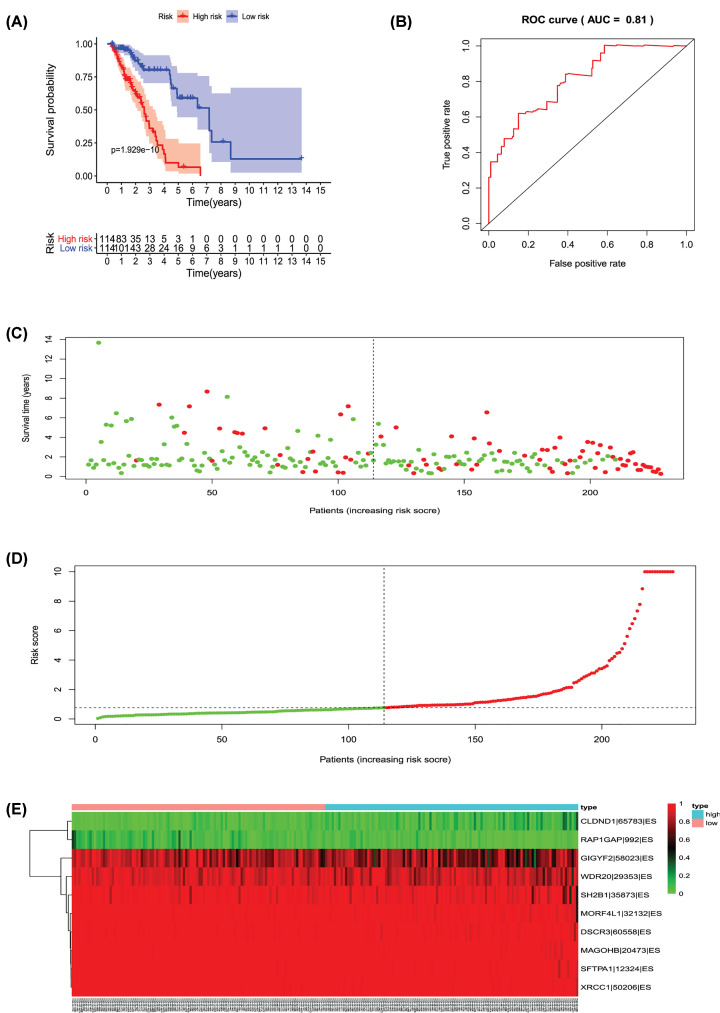
Analysis of the PI-ES The 10 prognosis-associated events of ES were selected by multivariate Cox regression analysis to construct the PI-ES model. (**A**) The risk curve of the PI for evaluating survival geriatric patients with LUAD stratified by the median PI value into a low-risk and high-risk groups. (**B**) Time-dependent receiver-operator characteristic curve of PI for predicting the tumor status. (**C**) PI value curve for geriatric patients with LUAD. (**D**) Survival conditions and survival time of GLAD patients distributed according to risk score (green dots represent survivors; red dots represent deaths). (**E**) Heat map indicating the relationship between the PSI value of the AS events and the risk score.

**Figure 11:**
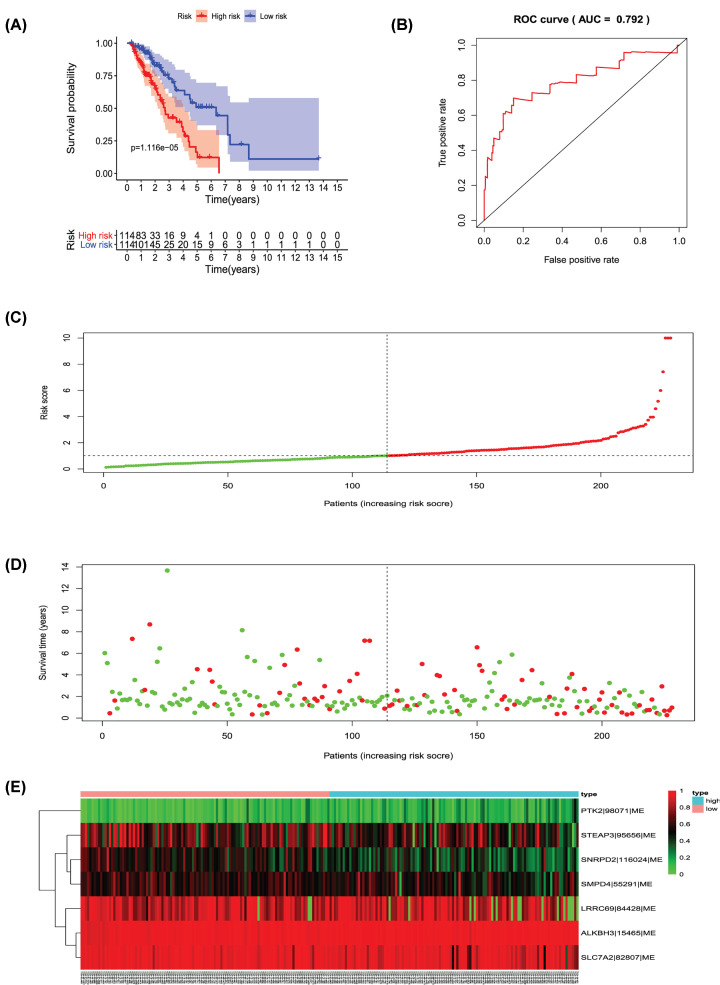
Analysis of the PI-ME The seven prognosis-associated events of ME were selected by multivariate Cox regression analysis to construct the PI-ME model. (**A**) The risk curve of the PI for evaluating the survival time of geriatric patients with LUAD stratified by the median PI value into low-risk and high-risk groups. (**B**) Time-dependent receiver-operator characteristic curve of PI for predicting the tumor status. (**C**) PI value curve for geriatric patients with LUAD. (**D**) Survival conditions and survival time of GLAD patients distributed according to risk score (green dots represent survivors; red dots represent deaths). (**E**) Heat map indicating the relationship between the PSI value of the AS events and the risk score.

**Figure 12:**
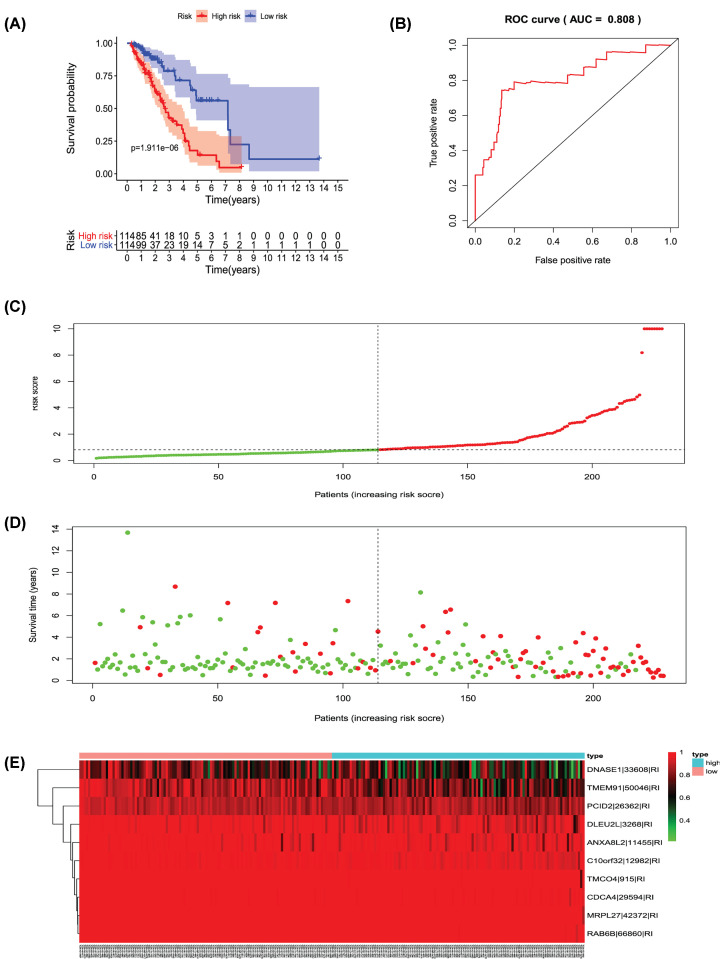
Analysis of the PI-RI The 10 prognosis-associated events of RI were selected by multivariate Cox regression analysis to construct the PI-RI model. (**A**) The risk curve of the PI for evaluating the survival time of geriatric patients with LUAD stratified by the median PI value into a low-risk and high-risk groups. (**B**) Time-dependent receiver-operator characteristic curve of the PI for predicting the tumor status. (**C**) PI value curve for geriatric patients with LUAD. (**D**) Survival conditions and survival time of GLAD patients distributed according to risk score (green dots represent survivors; red dots represent deaths). (**E**) Heat map indicating the relationship between the PSI value of the AS events and the risk score.

**Figure 13:**
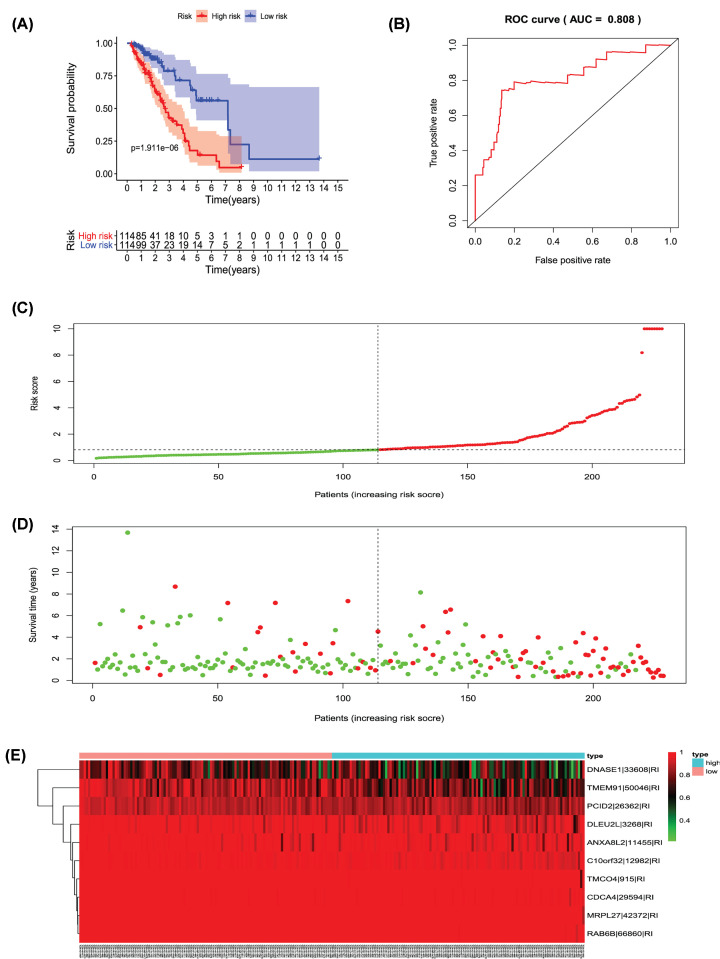
Analysis of the PI-ALL The ten prognosis-associated events of all types were selected by multivariate Cox regression analysis to construct the PI-ALL model. (**A**) The risk curve of PI to evaluate the survival time of geriatric patients with LUAD stratified by the median PI value into low-risk and high-risk groups. (**B**) Time-dependent receiver-operator characteristic curve of PI to predict the tumor status. (**C**) PI value curve for geriatric patients with LUAD. (**D**) Survival conditions and survival time of GLAD patients distributed according to risk score (green dots represent survivors; red dots represent deaths). (**E**) Heat map indicating the relationship between the PSI value of the AS events and the risk score.

**Figure 14:**
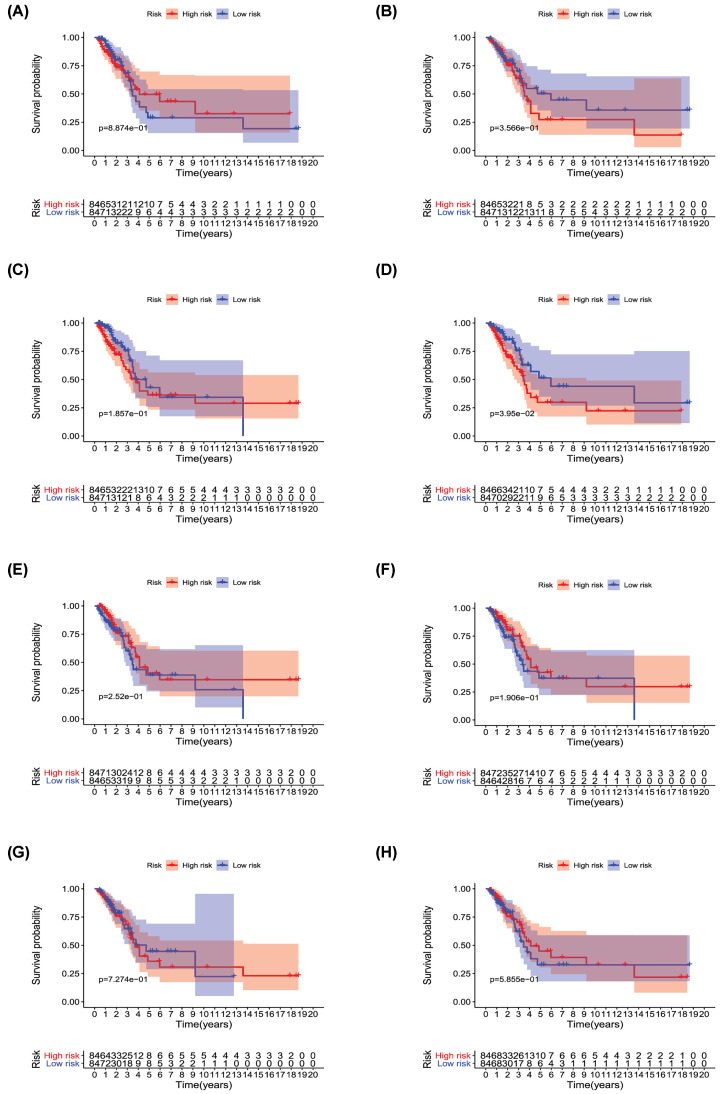
Non-elderly patients with lung adenocarcinoma data substituted into the PI model (**A**) PI-AA model (**B**) PI-AD model (**C**) PI-AP model (**D**) PI-AT model (**E**) PI-ES model (**F**) PI-ME model (**G**) PI-RI model and (**H**) PI-All model.

Univariate Cox regression analysis (*P*<0.001) was used to select significant survival associated with AS events for the construction of PI models. Then LASSO Cox analysis was conducted to eliminate interacting genes after cross-validation of minimum error (Figure 4A–H), and to screen significant survival-associated genes (Figure 5A–H). Genes with high survival correlations selected by LASSO regression were used to construct the PI models. The ROC curve analyses showed that eight models had predictive significance for prognosis. The PI model of AA events was the most effective at estimating the prognosis of geriatric patients with LUADs, with an AUC value of 0.87 (Figure 6B), followed by PI of all types and the PI of AD events, with an AUC value of 0.857 and 0.821, respectively (Figures 13B and 7B). Cases were then divided into high-risk and low-risk groups according to the median PI value. The results showed that all the PI models could achieve good stratification of the prognosis of the low- and high-risk groups (Figures 6–13). Kaplan–Meier curve analysis showed that the survival time of the low-risk group was significantly longer than the high-risk group (Figures 6A–13A). Patients with lower risk experienced a longer survival (*P*<0.001). According to the univariate and multivariate Cox regression analysis, PI models of AD splicing events showed the most significant survival time, with a value of 1.479E-13 (Figure 7A), followed by the PI of AA events and PI of all types of splicing events, with values of 2.053E-12 and 1.377E-11, respectively (Figures 6A and 13A). In all PI models, as the risk score increased, geriatric patients with LUAD were more inclined to experience a poorer prognosis (Figures 6C–13C). Heat maps were used to illustrate the relationship between AS events and the risk score. If the risk value positively correlated with the PSI value of the AS events, the AS event was considered as high-risk event (Figures 6E–13E). After substituting the data of non-elderly lung adenocarcinoma patients into the prognosis model, we found that there was no significant difference in survival between the high-risk group and low-risk group (Figure 14A–H).

### Survival-associated splicing factors-alternative splicing networks

**Figure 15:**
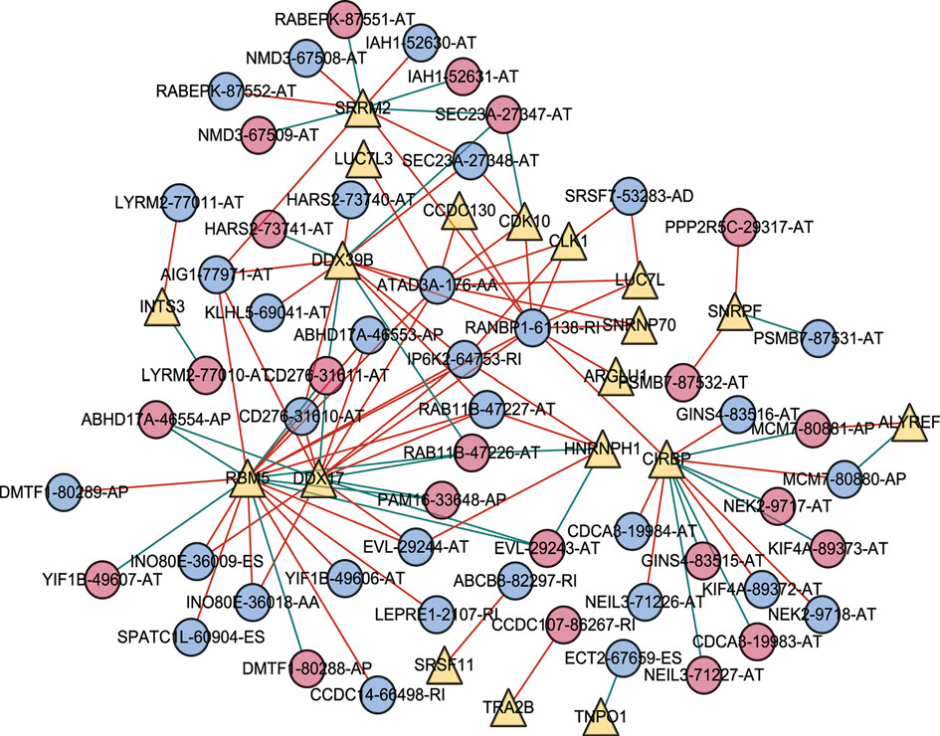
Correlation network between survival-associated splicing factors and splicing in geriatric patients with LUAD The PSI values of survival-related AS events are represented by red/blue dots. Survival-associated SFs are represented by yellow dots. The positive/negative correlations between expressions of the SFs and PSI values for AS are represented by red/green lines.

Previous studies have suggested that the dysregulation of SF plays a crucial role in tumorigenesis or progression [8]. Data relative to the mRNA expression of SFs in geriatric lung adenocarcinoma were downloaded from the TCGA database. Pearson’s correlation analysis was used to analyze the correlation between survival-related SFs and AS events with independent predictive significance (screening criteria: |correlation coefficient| > 0.6, *P*<0.001). A total of 19 SF and 54 AS events were selected by Pearson’s analysis. A correlation network was established using Cytoscape to examine the potential regulatory association between the SF and the survival-associated AS events (Figure 15). A shown in Figure 15, a single SF can be involved in regulating multiple AS events, and an AS event can be regulated by multiple SFs. Generally, AS events with high-risk were mainly negatively correlated with SFs, whereas AS events with low-risk were mainly positively correlated. However, some SFs are negatively correlated with low-risk AS events. For example, *SNRPF* showed a negative correlation with *PSMB7*-87531-AT but a positive correlation with *PSMB7*-87532-AT.

**Figure 16:**
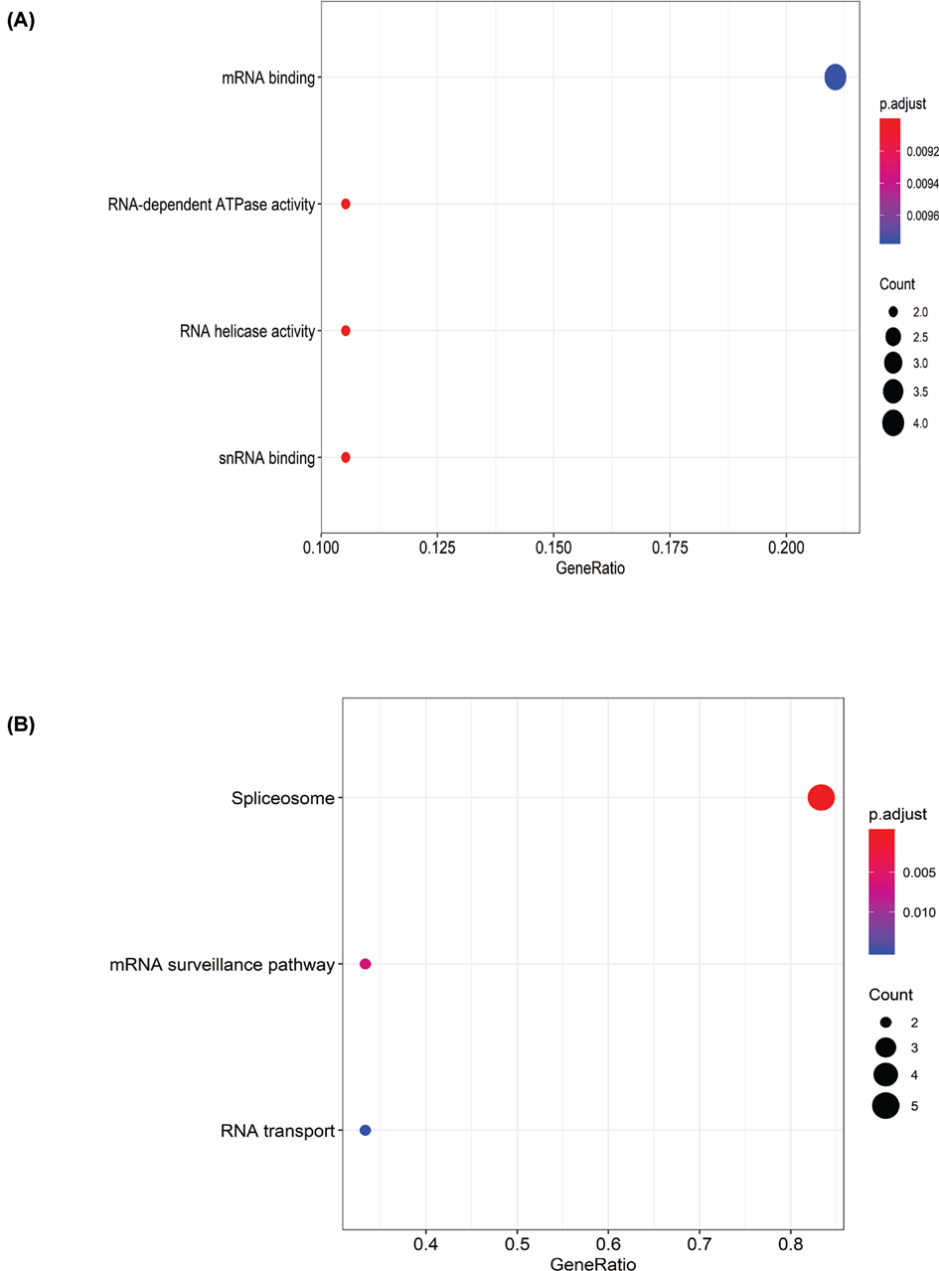
Biological function analysis (**A**) Terms identified by Gene Ontology (GO) analysis. The dot size represents the number of the enriched genes, and FDR values are shown by the color scale; FDR, false discovery rate. (**B**) Enrichment pathways identified by Kyoto Encyclopedia of Genes and Genomes (KEGG).

To investigate the potential biological function of the 19 SFs, we analyzed the biological pathways involved and identified enriched pathways using the R package. In GO terms, genes were mostly enriched in terms involving RNA-dependent ATPase activity, RNA helicase activity, snRNA binding, and mRNA binding (Figure 16A). Further, three KEGG pathways were enriched in the AS-SFs network, including the spliceosome, the mRNA surveillance pathway, and RNA transport (Figure 16B). We determined that the gene *DDX39B* was involved in seven biological functions (Table 2).

**Table 2:** The enrichment results in the alternative splicing- splicing factor regulatory network

Category	Description		Count	*P*-value	Gene ID
GO_Biology process	GO: 0008186	RNA-dependent ATPase activity	2	0.0004	*DDX17, DDX39B*
	GO: 0003724	RNA helicase activity	2	0.0007	*DDX17, DDX39B*
	GO: 0017069	snRNA binding	2	0.0007	*SNRNP70, DDX39B*
	GO: 0003729	mRNA binding	4	0.0011	*CIRBP, TRA2B, RBM5, LUC7L3*
KEGG pathway	hsa03040	Spliceosome	5	1.2747E-08	*SNRPF, SNRNP70, TRA2B, ALYREF, DDX39B*
	hsa03015	mRNA surveillance pathway	2	0.0020	*ALYREF, DDX39B*
	hsa03013	RNA transport	2	0.0075	*ALYREF, DDX39B*

Abbreviations: GO, Gene Ontology; KEGG, Kyoto Encyclopedia of Genes and Genomes.

## Discussion

Splicing of pre-mRNA is essential for the maturation of mRNAs, and it is an important step in regulating the expression of protein and genes. Abnormal AS in pre-mRNA splicing may lead to the development of tumors. Previous studies have revealed that SFs can regulate aberrant AS events that affect the occurrence and development of lung cancer. For example, Liu et al. showed that abnormal splicing of *BIN1* was controlled by serine and arginine-rich factor 1 (*SRSF1*) in NSCLC [32]. Lin et al. showed that *SRSF1* and *RBM4* had differential impact on *HIF-1α* in a CU element-dependent manner [33]. However, these studies were limited to a single splicing factor or splicing event and did not group the types of lung cancer evaluated. Our study focused on analyzing prognosis related AS events and SFs in elderly lung adenocarcinoma patients. We screened the SFs and AS events related to the prognosis of geriatric patients with LUAD and established eight PI models to analyze the risk score of different AS events. Then, we screened the SFs and AS events that were highly correlated with each other using Pearson correlation analysis. A total of 54 AS events and 19 SFs were selected to construct a correlation network that could help elucidate the potential mechanisms involved in the splicing pathway in GLAD.

The ROC curve analyses verified that all the models had a certain guiding significance for prognosis, and the PI model of AA events was the most significant. In the AA model, we analyzed 12 AS events closely related to the prognosis of GLAD, related genes mainly include *LIMD2, USP28, IL32*, and *ZC3H13*. LIM domain containing 2 (*LIMD2*) is a small LIM-only protein that has been revealed to promote tumor progression. A recent study showed that *LIMD2* serves as an oncogenic in NSCLC and was regulated by miR-34a and miR-124 [34,35]. Ubiquitin-specific proteases (*USP*) regulate physiological homeostasis of the ubiquitination process, a high level of *USP28* is related to poor overall survival and prognosis in NSCLC patients [35]. *IL32* and *ZC3H13* also play an important role in tumorigenesis and metastasis of lung adenocarcinoma [36,37]. These are consistent with our findings. AS is a complex and sophisticated process and SFs can regulate the aberrant AS events that affect the occurrence and development of lung cancer. Therefore, the correlation between SFs and AS events with independent prognostic value is worth studying.

In our study, a total of 19 SFs were included in the correlation network. We analyzed the regulation relationship between SFs and AS events, our findings may provide new insight for precise treatment and explain the potential molecular mechanism of GLAD. Different SFs often have a synergistic effect when regulating the same AS event. For example, *ATAD3A* is positively regulated by several SF such as *RBM5, DDX17, CDK-10, CLK1, CCDC130, LUC7L, SNRNP70*, and *LUC7L3*. Besides, one SF may positively or negatively affect different AS events. For example, *SRRM2* positively regulates *RANBP1*-61138-RI, whereas it negatively regulates *SEC23A*-27347-AT. In our network about GLAD, positive correlations between SFs and AS events were more common than negative ones. Generally, AS events with high-risk are mainly negatively correlated with SFs, whereas AS events with low-risk are mainly positively correlated. In other words, most of the SF in our study may delay the progression of GLAD except *SNRPF, ALYREF*, and *TNPO1*.

Several SFs in key nodes were frequently related to splicing events in GLAD, mainly including *DDX39B, DDX17, SRRM2, CIRBP*, and *RBM5*. Prior studies have shown that these SF are closely related to tumor formation. Overexpression of *DDX39B* enhances cell proliferation and global translation to promote tumor formulation [38]. *DDX17* promotes the formation of hepatocellular carcinoma by inhibiting *Klf4* transcriptional activity [39]. *SRRM2* is a main component of the spliceosome, and mutation in *SRRM2* is associated with the predisposition of papillary thyroid carcinoma [40]. Abnormal expression of *CIRBP* is involved in the progression and migration of nasopharyngeal carcinoma and bladder cancer [41,42]. Besides, *RBM5* can be acted as a tumor suppressor [43], and it inhibits the formation of lung adenocarcinoma through several apoptotic signaling pathways [44]. It was consistent with our study findings that *RBM5* could up-regulate several low-risk AS events and down-regulate high-risk AS events. However, previous studies did not discuss the role of *DDX39B, DDX17, SRRM2*, and *CIRBP* in GLAD. In our analyses, these SF were found to affect tumor prognosis by regulating several AS events. For example, *DDX39B* positively regulates the *ATAD3A*-176-AA, whereas negatively regulates the *RAB11B*-47226-AT, and GO function terms and KEGG pathway analysis showed that *DDX39B* was involved in seven biological functions. *DDX17* positively regulates the *AIG1*-77971-AT, whereas negatively regulates the *RAB11B*-47227-AT. Furthermore, we found that *DDX39B, DDX17*, and *RBM5* were simultaneously positively regulated the *ATAD3A*-176-AA. *ATAD3A* is a kind of mitochondrial enzyme, and it is a low-risk gene associated with AA event [45]. The deregulation of *ATAD3A* is crucial in the tumor microenvironment because it promotes tumor metastasis [46]. As such, *ATAD3A* is a promising drug target in the treatment of GLAD. However, there are few studies on *ATAD3A* in lung adenocarcinoma, the clinical significance of targeting *ATAD3A* in the treatment of GLAD deserves further exploration and demonstration. Therefore, our study may highlights a possible mechanism in GLAD tumorigenesis.

The results of the GO analysis indicated that the genes were mainly involved in RNA-dependent ATPase activity, RNA helicase activity, snRNA binding, and mRNA binding. Furthermore, three KEGG pathways were enriched in the AS-SFs network, including the spliceosome, the mRNA surveillance pathway, and RNA transport. It is worth noting that DDX39B was involved in seven biological functions. The AS events generated from these genes may affect the development of GLAD by interfering with the above biological processes and pathways.

In conclusion, we assessed the prognostic value of survival-related AS events in GLAD and established eight PI models with high prognostic values. The regulatory network and enrichment analyses revealed the distinct relationship between AS and SFs. The process of these specific regulatory mechanisms in spliceosomes may serve as crucial starting points for further exploration of splicing events in GLAD.

## Data Availability

The datasets used and analyzed during the current study are available from the online tools.

## Abbreviations

AA: alternate acceptor site
AD: alternate donor site
AP: alternate promoter
AS: alternative splicing
AT: alternate terminator
AUC: area under the curve
ES: exon skip
GDC: Genomic Data Commons
GLAD: geriatric lung adenocarcinoma
GO: Gene ontology
HR: hazard ratio
K-M: Kaplan–Meier curve
KEGG: Kyoto Encyclopedia of Genes and Genomes
LASSO: least absolute shrinkage and selection operator
LUAD: lung adenocarcinoma
ME: mutually exclusive exons
NSCLC: non–small cell lung carcinoma
PI: prognostic index
PSI: percent-sliced-in
RI: retained intron
ROC: receiver-operator characteristic
SF: splicing factor
TCGA: The Oncology Genome Atlas
